# Down-Regulating the Expression of miRNA-21 Inhibits the Glucose Metabolism of A549/DDP Cells and Promotes Cell Death Through the PI3K/AKT/mTOR/HIF-1α Pathway

**DOI:** 10.3389/fonc.2021.653596

**Published:** 2021-05-11

**Authors:** Ye Sun, Wenjun Liu, Qiuyu Zhao, Ruiqi Zhang, Jianbo Wang, Pengyu Pan, Hai Shang, Chunying Liu, Chun Wang

**Affiliations:** ^1^ Department of Cell Biology, College of Integrated Chinese and Western Medical, Liaoning University of Traditional Chinese Medicine, Shenyang, China; ^2^ Key Laboratory of Environmental Pollution and Microecology of Liaoning Province, Shenyang Medical College, Shenyang, China; ^3^ Teaching and Experimental Center, Liaoning University of Traditional Chinese Medicine, Shenyang, China; ^4^ Key Laboratory of Ministry of Education for Traditional Chinese Medicine (TCM) Viscera-State Theory and Applications, Liaoning University of Traditional Chinese Medicine, Shenyang, China; ^5^ Northeast Yucai School, Shenyang, China; ^6^ Cancer Hospital of China Medical University, Liaoning Cancer Hospital & Institute, Shenyang, China

**Keywords:** non-small cell lung cancer, cisplatin resistance, miRNA-21, PI3K/AKT/mTOR, glycolysis

## Abstract

miRNA-21 is a single-stranded non-coding RNA that is highly expressed in a variety of tumor cells. It participates in tumor cell proliferation, metabolism, metastasis, and drug resistance. Here, we tested the potential mechanism of miRNA-21 in cisplatin-resistant non-small cell lung cancer A549/DDP (human lung adenocarcinoma drug-resistant cell line) cells. A549 and A549/DDP RNAs were sequenced to show that miRNA-21 was highly expressed in the latter, and this was verified by qRT-PCR. In addition, we found that miRNA-21 combined with cisplatin can significantly inhibit glycolysis and glycolysis rate-limiting enzyme protein expression in A549/DDP cells. We also found that miRNA-21 combined with cisplatin can promote A549/DDP cell death. Further investigations showed that miRNA-21 combined with cisplatin caused excessive inactivation of the pI3K/AKT/mTOR/HIF-1α signaling pathway in cisplatin-resistant A549/DDP cells. Hence, reduction of the expression of miRNA-21 in combination with cisplatin chemotherapy may effectively improve the therapeutic effect on patients with non-small cell lung cancer, and this may provide a theoretical basis for the treatment of this disease.

## Introduction

Although great efforts have been made in the treatment of cancer, lung cancer is still one of the most fatal malignant kinds of cancers (1, 2). At present, the best treatment for lung cancer is surgery combined with chemotherapy drugs (3). Chemotherapy based on cisplatin has greatly improved the prognosis and quality of life of patients with lung cancer. Cisplatin is a kind of chemotherapy drug that can promote DNA damage and induce apoptosis (4–6). However, the recurrence rate of cancer is very high after cisplatin treatment (7). Emergence of cisplatin resistance limits its efficacy in lung cancer patients; this has always been a difficult problem. Therefore, it is of great importance to find new drug combinations to improve the sensitivity of lung cancer cells to cisplatin.

In recent years, studies have confirmed that the PI3K/Akt/mTOR signaling pathway is abnormally active in human tumors. This pathway regulates the occurrence and development of tumor cells, as well as their growth and proliferation, material metabolism, cytoskeleton remodeling, invasion, metastasis, and other physiological activities (8). The failure to activate the PI3K/Akt/mTOR-mediated death process, including apoptosis or autophagic death, is a major mode of drug resistance in tumor cells (9). In addition, tumors highly use the aerobic glycolysis pathway to provide energy for themselves, creating an acidic microenvironment to enhance tumor growth. This is also an important mode of tumor drug resistance (10). Therefore, a better understanding of the PI3K/Akt/mTOR pathway would be helpful for better treatment of tumors.

Sequencing of human and other mammalian genomes found that more than 90% of gene sequences can transcribe RNA, but 80-90% of RNA is non protein coding RNA (ncRNA), and only 2% of RNA is protein coding RNA (11–13). Therefore, most transcripts are called non-coding RNAs (ncRNAs). MicroRNA (miRNA) is one of the important members of the non-coding RNA family. miRNA is related to the occurrence and development of human cancer, and plays a role by activating the targets of oncogene pathways or by interacting with cancer-related protein coding genes (14). In addition, miRNA differential expression profiles are closely related to the diagnosis, staging, progression, prognosis, and treatment of tumors (15). miRNA-21 is one of the miRNAs, and is significantly increased in glioblastoma, hepatocellular carcinoma, ovarian cancer, colorectal cancer, and lung cancer (16–20). A recent study has confirmed that inhibition of miRNA-21 expression can increase the apoptosis and necrosis of non-small cell lung cancer (NSCLC) A549 cells induced by 5-fluorouracil. A further study showed that the target of miRNA-21 sensitization chemotherapy was achieved through the PTEN pathway (21). In addition, it has been reported that inhibition of miRNA-21 expression can reduce the activity of the PTEN pathway, thereby reducing the glycolysis level in pancreatic stellate cells and inhibiting cell invasion and migration (22). However, whether miRNA-21 is related to NSCLC resistance is unclear.

In this study, RNA sequencing (RNA-Seq) was used to evaluate the difference in transcriptome level between a cisplatin-sensitive lung cancer cell line (A549) and a cisplatin-resistant lung cancer cell line (A549/DDP). RNA-Seq was also used to find differences in glucose metabolism-related mRNA and miRNA-21 expression levels that could be related to the formation of drug resistance. We explored whether miRNA-21 is involved in the regulation of A549/DDP cisplatin resistance. We also investigated whether miRNA-21’s regulation of cisplatin resistance is related to tumor cell glucose metabolism.

## Materials and Methods

### Reagents and Antibodies

The main reagents used in this study included cisplatin (Sigma Co., Ltd., MO, USA, Cat No. PHR1624), a human glucose ELISA kit (Cat No. m1063205-C), a PyC (pyruvic acid, Cat No. m1061454-C) ELISA kit, and a human LA (lactic acid, Cat No. m1037915-C) ELISA kit (Shanghai Enzyme-linked Biotechnology Co., Ltd., Shanghai, China). The primary antibodies include mouse monoclonal anti-PI3K (1:1000, Cat No. 67071-1-1-Ig), rabbit polyclonal anti-AKT (1:1000, Cat No. 10176-2-AP), mouse monoclonal anti-p-AKT (Ser473, 1:1000, Cat No. 66444-1-Ig), rabbit polyclonal capase 3/p17/p19 (1:1000, Cat No. 19677-1-AP), rabbit polyclonal Bcl2 (1:1000, Cat No. 26593-1-AP), rabbit polyclonal BAX (1:1000, Cat No. 50599-2-Ig), goat anti-mouse IgG (1:5000, H+L, Cat No. SA00001-1) and goat anti-rabbit IgG (1:5000, H+L, Cat No. SA00001-2) (Proteintech Group Inc., Rosemont, IL, USA), rabbit polyclonal anti-p-PI3K (P85) (1:1000, Chengdu ZenBio Antibody Co., Ltd., Chengdu, China, Cat No. 530854), rabbit polyclonal p-Akt (1:1000, Thr308, Cat No. 13038S), rabbit polyclonal mTOR(1:1000, Cat No. 13038S), rabbit monoclonal p-mTOR (1:1000, Ser2448, Cat No. 5536S), rabbit monoclonal HIF-1α (1:1000, Cat No. 36169S), rabbit monoclonal c-Myc (1:1000, Cat No. 5605S), rabbit monoclonal HK2 (1:1000, Cat No. 2867S), rabbit monoclonal PKM2 (1:1000, Cat No. 4053S), rabbit monoclonal Atg7 (1:1000, Cat No. 8558S), and rabbit monoclonal LC3B (1:1000, Cat No. 3868S) (Cell Signaling Technology Inc., Danvers, MA, USA).

### Cell Lines and Cell Culture

Human lung adenocarcinoma cell line A549 and human lung adenocarcinoma cisplatin-resistant cell line A549/DDP were purchased from the Beijing Dingguo Changsheng Biotechnology Company, Ltd. (Beijing, China). The A549 cells were cultured in Dulbecco’s modified Eagle’s medium/high glucose (Hyclone, Logan, UT, USA, Cat No. SH30228.01), containing 10% fetal bovine serum (SciTech, MS, USA, Cat No. S-FBS-500), 100 units/mL penicillin, and 100 μg/mL streptomycin solution (Hyclone, Logan, UT, USA, Cat No. SV30010). The A549/DDP cells were cultured in McCoy’s 5A medium (Genview, FL, USA, Cat No. GM3109-500ML) containing 10% fetal bovine serum (SciTech, Oxford, MS, USA, Cat No. S-FBS-500), 100 units/mL penicillin, and 100 μg/mL streptomycin solution (Hyclone, Logan, UT, USA, Cat No. SV30010). The drug resistance of A549/DDP cells was maintained in complete culture medium containing 2 μg/mL cisplatin without penicillin-streptomycin antibiotics, and the drug was withdrawn two weeks before the experiment. Two cell lines were cultivated at 37°C in a 5% CO_2_ incubator.

### RNA Extraction and RNA Quality Analysis

The total RNA of A549 and A549/DDP cells were extracted with RNAiso Plus reagent (Takara Bio Inc., Dalian, China, Cat No. 9108), and the quantity and quality of total RNA in each sample were determined by NanoDrop ND-1000 spectrophotometer (Thermo Fisher Scientific Inc., Waltham, MA, USA). Then the RNA samples were sent to KangChen Bio-tech Co., Ltd. (Shanghai, China) for microarray experiments and data analyses.

### RNA Library Construction and RNA-Seq Assay

The total RNA content in each sample ranged from 1 to 2 μg, and mRNA enrichment was performed using the NEB Next^®^ Poly(A) mRNA Magnetic Isolation Module kit (New England Biolabs Inc., Ipswich, MA, USA, Cat No. E7490S). Then the library was constructed using a KAPA Stranded RNA-Seq Library Prep Kit (Roche Inc., Boston, MA, USA, Cat No. KK8401). After denaturation with 0.1 M NaOH, the single-stranded DNA was diluted to 8 pM and then amplified *in situ* with a TruSeqSR Cluster Kit v3-cBot-HS (Illumina Inc., San Diego, CA, USA, Cat No. PE-401-3001). The end of the resulting fragment was sequenced for 150 cycles on an Illumina HiSeq4000 sequencer.

### Functional Enrichment Analysis of mRNA

First of all, we conducted Gene Ontology (GO) enrichment analysis on differentially expressed transcripts and used Fisher’s exact test to find out which specific functional items were most related to a group of differentially expressed genes. In addition, we also used KEGG (Kyoto Encyclopedia of Genes and Genomes) pathway enrichment analysis, mainly to compare the differentially expressed mRNAs among samples with the biological pathway resources of the KEGG database (http://www.genome.ad.jp/kegg/). By comparing and enriching these differentially expressed mRNAs with known functional items, we can obtain the relationship between differentially expressed genes and specific functional items. The critical *p*-value was 0.05; the lower the *p*-value, the more significant the correlation was.

### Cell Transfection

Cells at a density of 2×10^5^ cells/well were plated into 6-well plates and cultured for 18 h. The density of cells reached more than 70%. miRNA-21 NC plasmid (4 μg, target sequence: TTCTCCGAACGTGTCACGT) or miRNA-21 sponge plasmid (4 μg, target sequence: TCAACATCAGTCTGATAAGCTA) (GenePharma Co., Ltd., Shanghai, China) was added into 20 μL basic McCoy’s 5A medium, and 2 μL GoldenTran DR Reagent (Golden Trans Technology Co., Ltd., Changchun, China, Cat No. PE-401-3001) was then added into the same solution. After 10 min of incubation at room temperature, the plasmid solution was mixed. The complex was added into the 6-well plate. After 8 h, the culture medium was changed. Then, 24 h later, cisplatin was added to co-treat the cells for the next 24 h. Finally, cell culture medium was collected or cells were lysed to collect total protein or RNA.

### Determination of Glucose, Pyruvic Acid, and Lactic Acid

The A549/DDP cells were plated at 4×10^5^ cells per well and cultured in 6-well plates for 18 h. Then the A549/DDP cells were treated with miRNA-21 NC, cisplatin, miRNA-21 sponge, or miRNA-21 sponge combined with cisplatin for 48 h. After treatment, the cell culture medium was collected, and the levels of glucose, pyruvic acid, and lactic acid in the supernatant were determined with the Human Glucose ELISA kit (Cat No. m1063205-C), Human PyC ELISA kit (Cat No. m1061454-C), and the Human LA ELISA kit (Cat No. m1037915-C) (MIBIO, Shanghai, China); the experimental process followed the manufacturer’s protocol.

### Determination of Pyruvate Kinase (PK) and Lactate Dehydrogenase (LDH) Enzyme Activity

One milliliter of the extract was added to 5×10^6^ cells of A549 and A549/DDP, and cell disruption was performed in a DHS high-throughput tissue grinder. The reaction conditions were 1500 rpm and 30 sec. The PK activity test kit (Solarbio Science & Technology Co., Ltd., Beijing, China, Cat No. BC0545) and the LDH activity test kit (Solarbio Science & Technology Co., Ltd, Beijing, China, Cat No. BC0685) were used to determine the activity of PK and LDH in cells. The experimental process followed the manufacturer’s protocol.

### Glycolysis Stress Test

XF 96-well microplates were used to inoculate 8×10^3^ cells at a total cell suspension volume of 100 µL. and the cells were then incubated at 37°C with 5% CO_2_ for 24 h. Thereafter, the culture medium was removed, and the cells were washed with 2 mmol/L glutamine and incubated for 1 h at 37°C without CO_2_. In the next step, the XF glycolysis stress kit (Seahorse Bioscience, Billerica, MA, USA, Cat No. 103020-100) was used to add the following substances to each well in turn: glucose (300 µM), oligomycin (72 nM), and 2-deoxy-D-glucose (2-DG, 1500 µM) to measure the extracellular acidification rate (ECAR). Finally, the XF96 Extracellular Flux Analyzer (Seahorse Bioscience, Billerica, MA, USA) was used to measure the ECAR.

### Real-Time PCR (qRT-PCR)

Total RNA was extracted from cells with RNAiso Plus reagent (Takara Bio Inc., Dalian, China, Cat No. 9108). For miRNA-21, the cDNA was synthesized with miRNA-specific reverse transcription primers, and qRT-PCR was performed by the SYBR green dye method using the Hairpin-it™ miRNA qRT-PCR Quantitation kit (GenePharma Co., Ltd., Shanghai, China, Cat No. E01006) according to the manufacturer’s protocol. The qRT-PCR conditions for miRNAs were as follows: 3 min at 95°C, followed by 40 cycles of 95°C for 12 sec, and 62°C for 40 sec. *U6* gene expression was an internal control. The first-strand cDNAs of PKM2 and LDHA were synthesized with HiScript II Q Select RT SuperMix for the qRT-PCR kit (Vazyme Biotech Co., Ltd., Nanjing, China, Cat No. R232-01). The relative mRNA expression levels of PKM2 and LDHA were measured with the qRT-PCR SYBR Green Master Mix kit (Vazyme Biotech Co., Ltd., Nanjing, China, Cat No. Q111-03), and β-actin gene expression was regarded as an internal control. Data were analyzed using the 2^-ΔΔCT^ method.

### Determination of Fluorescence Spots From LC3

The cells grown on the 6-well plate were fixed in 4% paraformaldehyde (Solarbio Co., Ltd., Beijing, China, Cat No. P1110) for 10 min, washed with PBS three times (each time for 5 min) (Hyclone, Logan, UT, USA, Cat No. SH30256.01), infiltrated with 1% Triton X-100 solution (Beyotime Co., Ltd., Shanghai, China, Cat No. ST795) for 30 min, and treated with 1% glycine solution (Beyotime Co., Ltd., Shanghai, China, Cat No. ST085) at room temperature for 1 h. Next, the samples were incubated with microtubule-associated LC3-II antibody (1:100, Proteintech, Chicago, IL, USA, Cat No. 18725-1-AP) overnight at 4°C, washed three times in PBS with 0.01% Triton X-100, and then treated with secondary antibody (FITC-bound rabbit IgG, 1:200, ZSGB BIO, Beijing, China, Cat No. ZF-0311) at 37°C. The cells were further stained with DAPI and observed under a fluorescence microscope (Zeiss, Germany).

### Western Blot Analysis

Treated cells were washed with precooled PBS solution once (Hyclone, Logan, UT, USA, Cat No. SH30256.01) and lysed with RIPA lysate (Cat No. P0013B) PMSF (the final concentration was 1 mM, Cat No. ST505) (Beyotime Co., Ltd., Shanghai, China). BCA protein assay (Leagene, Beijing, China, Cat No. PT0001) was used to measure the protein concentration. We loaded 50 μg protein onto an SDS-PAGE gel (Dingguo Changsheng Co., Ltd., Beijing, China, Cat No. WB-0211), and the electrophoresis conditions were set at 80 V for 20 min, followed by 120 V until the protein loading buffer reached the bottom. Then, the proteins were transferred to PVDF membrane (Millipore MA, USA, Cat No. IPVH00010) under 300 mA for 2 h. Five percent milk was used to block membranes overnight, diluted primary antibody and secondary antibody were incubated with membranes at room temperature for 1 h each, and the protein bands were exposed with enhanced ECL chemiluminescence reagent (Vazyme Biotech Co., Ltd., Nanjing, China, Cat No. E412-01/02).

### Statistical Analysis

Data were expressed using mean ± SD. The differences between the two groups were analyzed by two-tailed Student’s *t*-tests. An analysis of variance (ANOVA) test was used to compare multiple groups of data to determine the differences among groups. P < 0.05 was chosen to indicate a statistically significant difference. SPSS software 19.0 was used for statistical analysis.

## Results

### Sensitivity to Cisplatin of A549 Cells and A549/DDP Cells

**Figure 1:**
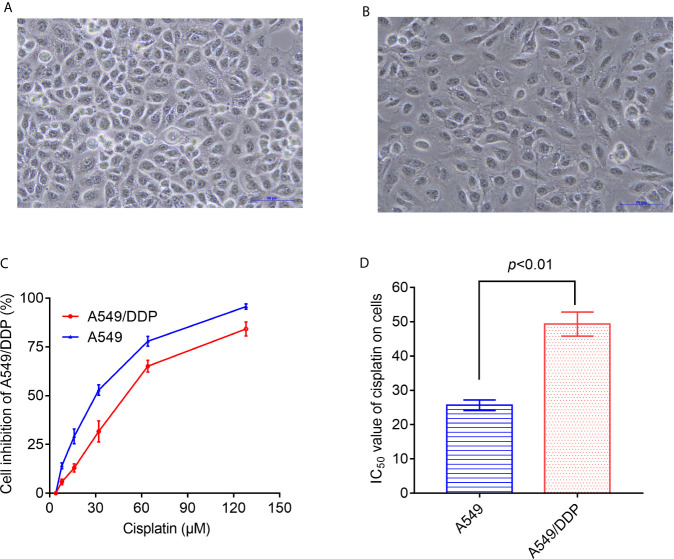
Differences in morphology and sensitivity to cisplatin between A549 and A549/DDP. Morphological observation of A549 and A549/DDP under phase contrast microscope **(A, B)**. Detection of inhibition rate of cisplatin on A549 and A549/DDP **(C)**. IC_50_ values of cisplatin in A549 and A549/DDP cells **(D)**. The experiments were repeated at least three times, and results were expressed as mean ± S.D.

As shown in **Figures 1A, B**, under the inverted microscope, the cell volume and cell spacing of A549/DDP cells were significantly increased compared with A549 cells. The IC_50_ values of cisplatin in A549 cells and A549/DDP cells were different. The drug resistance of A549/DDP cells was significantly higher than that of A549 cells (P < 0.05) (**Figures 1C, D**).

### RNA-Seq Shows a Huge Difference in mRNA Between A549 Cells and A549/DDP Cells

**Figure 2:**
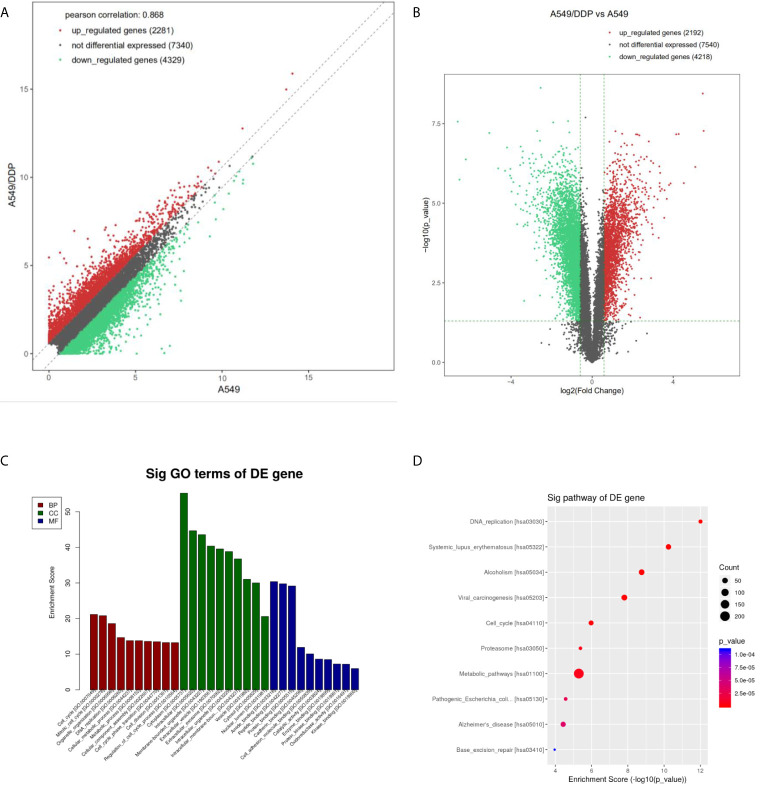
Differential expression and enrichment analysis of mRNA in A549 cells and A549/DDP cells. The mRNA levels of A549 and A549/DDP cells were calculated by **(A)** scatter plot and **(B)** volcano plot, respectively. The red dots represent the up-regulated mRNAs in A549/DDP cells, the green dots represent the down-regulated mRNAs in A549/DDP cells, and the gray dots represent the unchanged mRNAs. **(C)** GO and **(D)** KEGG were used for enrichment analysis of differentially expressed mRNAs in A549 and A549/DDP cells.

Differentially expressed mRNA was screened by an FDR algorithm and log_2_FC (FDR < 0.05 and |log_2_FC| > 1) using scatter plots and volcano plots. Compared with A549 cells, the number of up-regulated, differentially expressed mRNAs was approximately 2,200, while about 4,300 differentially expressed mRNAs were down-regulated (**Figures 2A, B**).

GO analysis showed that compared with A549 cells, the molecular functions of A549/DDP cells were differently changed: cell cycle, DNA replication, metabolic process, and cellular components had significant changes in cell composition. Biological processes had significant changes mainly related to binding and enzyme activity (**Figure 2C**). KEGG annotation results showed that compared with A549 cells, the expression levels of related genes such as those of the cell cycle pathway, DNA replication pathway, and cell metabolism pathway in A549/DDP cells were significantly up-regulated (**Figure 2D**).

### A549/DDP Cells Have Higher Glucose Metabolism Levels Than A549 Cells

**Figure 3:**
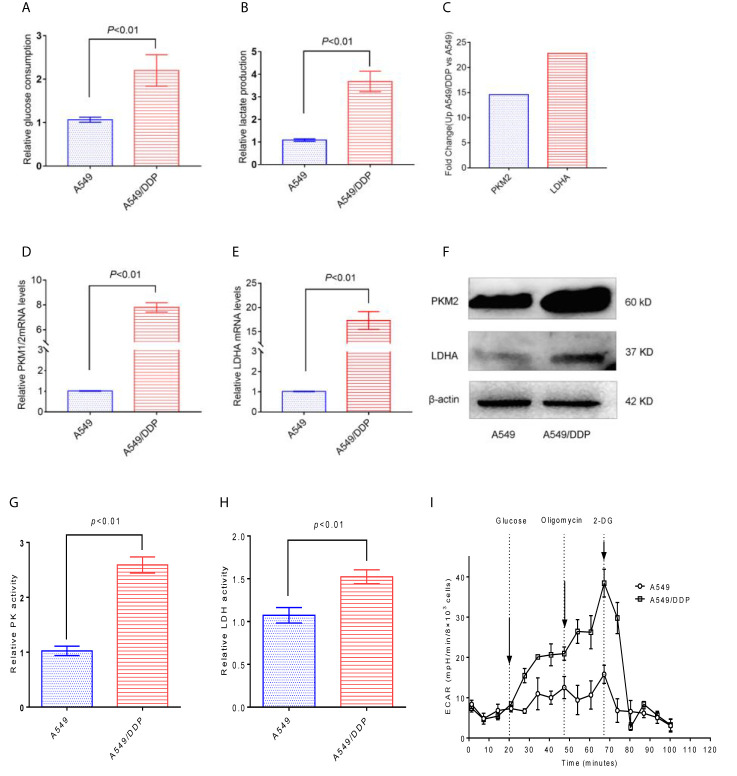
The glycolysis level of A549/DDP cells increased. **(A)** Glucose consumption and **(B)** lactate production were measured in A549 cells and A549/DDP cells. **(C)** KEGG results showed the expression of PKM2 and LDHA in A549 cells and A549/DDP cells. **(D, E)** qRT-PCR assay for the expression of glucose metabolizing enzymes PKM2 and LDHA in A549 cells and A549/DDP cells. The expression of glucose metabolizing enzymes PKM2 and LDHA increased in A549 cells and A549/DDP cells, as shown by Western blot analysis **(F)**. Determination of the activity of glucose-metabolizing enzymes PK and LDH in A549 cells and A549/DDP cells **(G, H)**. Determination of glycolytic stress in A549 cells and A549/DDP cells **(I)**. The results are presented as mean ± S.D; the experimental results are presented in triplicate. PKM2, pyruvate kinase M2; LDHA, lactate dehydrogenase A.

Compared with A549 cells, A549/DDP cells have significantly higher glucose consumption and lactate production (**Figures 3A, B**). Through the KEGG results, we found that the PKM2 and LDHA of A549/DDP cells increased 14- and 23- fold (**Figure 3C**), respectively. In addition, the qRT-PCR results verified that PKM2 and LDHA in A549/DDP cells were up-regulated by 7 and 17 times (**Figures 3D, E**), respectively. Then, the expression levels of the glucose metabolizing enzymes PKM2 and LDHA in A549 cells and A549/DDP cells were further analyzed by western blot technology, and it was found that these two enzymes were significantly increased in A549/DDP cells (**Figure 3F**). The protein expression levels and enzyme activity levels of glucose-metabolizing enzymes in A549 cells and A549/DDP cells were further analyzed using western blot technology and ELISA methods. The results showed that the protein expression levels of PKM2 and LDHA, and the enzyme activity levels of PK and LDH all increased significantly in A549/DDP cells (**Figures 3G, H**). Finally, by measuring the ECAR, we also found a significant increase in the glycolysis level of A549/DDP (**Figure 3I**).

### miRNA-21 Is Highly Expressed in A549/DDP Cells

**Figure 4:**
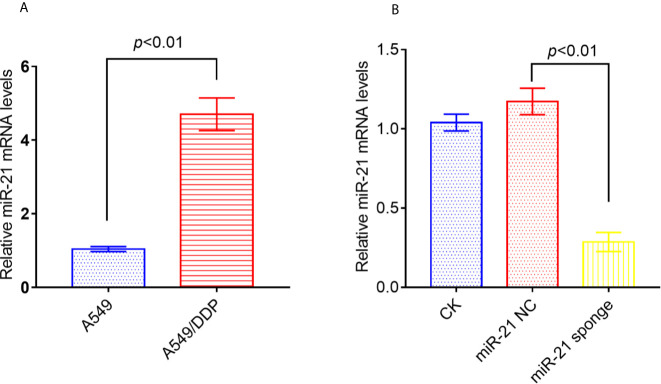
The expression of miRNA-21 increased in A549/DDP cells, and the transfection of miRNA-21 sponge inhibited the expression of miRNA-21. The expression of miRNA-21 was measured in A549 cells and A549/DDP cells **(A)**. Transfection of miRNA-21 into A549/DDP cells can inhibit the expression of miRNA-21 **(B)**. The experiments were repeated at least three times and results were expressed as mean ± S.D.

Studies have reported that miRNA-21 is highly expressed in A549/DDP cells compared to A549 cells. We confirmed by qRT-PCR that miRNA-21 expression is up-regulated in A549/DDP cells by 5-fold (**Figure 4A**). In addition, either miRNA-21 NC or miRNA-21 sponge was transfected into A549/DDP cells, and we found that the expression of miRNA-21 was significantly reduced in A549/DDP cells transfected with miRNA-21 sponge (**Figure 4B**).

### Down-Regulating the Expression of miRNA-21 Can Inhibit the Level of Glucose Metabolism in A549/DDP Cells and Enhance Their Sensitivity to Cisplatin

**Figure 5:**
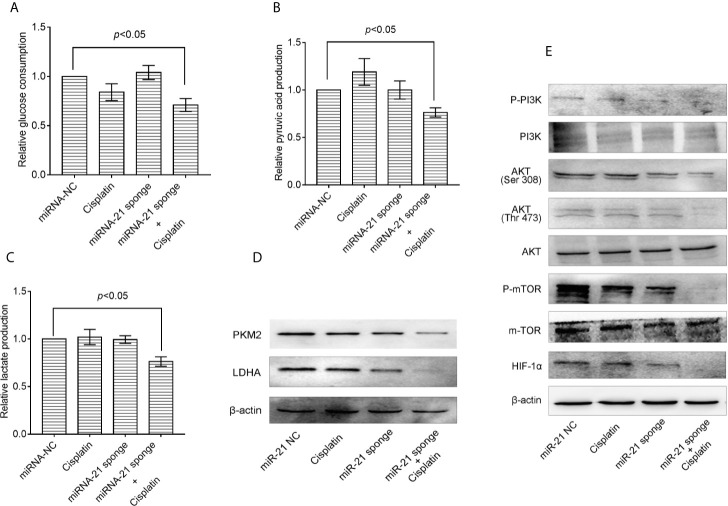
Combination of miRNA-21 and cisplatin inhibits glycolysis in A549/DDP cells through the PI3K/AKT/mTOR/HIF-1α signaling pathway. **(A)** Glucose consumption, **(B)** pyruvate production, and **(C)** lactate production were measured with miRNA-NC or miRNA-21 combined with cisplatin (IC_20_) in A549/DDP cells. Western blot was used to determine the expression of **(D)** glucose metabolizing enzymes (PKM2 and LDHA) and **(E)** PI3K/AKT/mTOR/HIF-1α pathway enzymes under miRNA-NC or miRNA-21 combined with cisplatin (IC_20_) in A549/DDP cells. The experiments were repeated at least three times and results were expressed as mean ± S.D. PI3K, Phosphoinositide 3-kinase; AKT, AKT serine/threonine kinase; mTOR, mechanistic target of rapamycin; p, phosphorylated; HIF-1α, hypoxia-inducible factor-1α.

Co-treatment of A549/DDP cells with miRNA-21 sponge combined with cisplatin can attenuate glucose consumption (**Figure 5A**), pyruvate production (**Figure 5B**), lactate production (**Figure 5C**), and PKM2 and LDHA expression levels (**Figure 5D**). Further studies found that the miRNA-21 sponge combination and cisplatin can down-regulate the expression levels of p-PI3K, p-AKT (Thr 308), p-AKT (Ser 473), m-TOR, and HIF-1α. As shown by a CCK-8 kit, down-regulation of miR-21 can significantly enhance the sensitivity of A549/DDP cells to cisplatin (**Figure 5E**).

### Down-Regulation of miRNA-21 Promotes Cell Death of A549/DDP Cells

**Figure 6:**
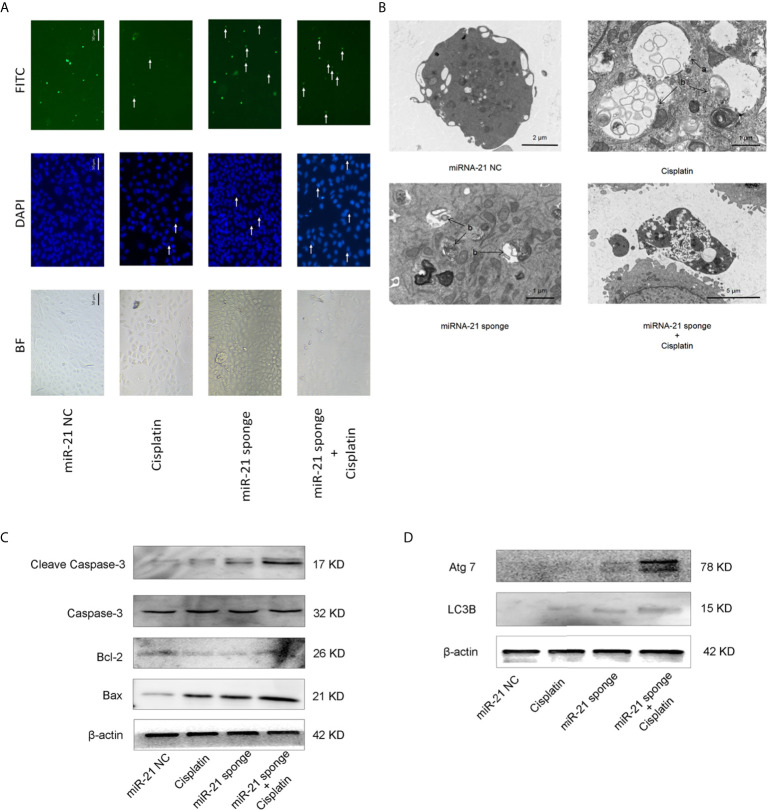
miRNA-21 combined with cisplatin promotes cell death in A549/DDP cells. **(A)** The effects of miRNA-21 combined with cisplatin on autophagy and apoptosis of A549/DDP cells were observed under a common microscope and a fluorescence microscope. In the DAPI field of vision, the white arrow marks the nuclear fragments during apoptosis. In the FITC field of vision, the white arrow marks the autophagosome **(B)** The effects of miRNA-21, combined with cisplatin, on autophagy, apoptosis, and the necrosis of A549/DDP cells were observed by transmission electron microscopy. In Figure **(B)**, a: autophagosome and b: autophagosome. Western blot was used to detect **(C)** the expression of autophagy and **(D)** the expression of apoptosis-related proteins in A549/DDP cells induced by miRNA-21, combined with cisplatin.

As shown in **Figure 6A**, when miRNA-21 combined with cisplatin intervened in A549/DDP cells, the autophagy of A549/DDP cells was significantly enhanced. LC3B spots bound with FITC secondary antibody were observed under a fluorescence microscope, and apoptotic bodies were observed by DAPI staining. In addition, transmission electron microscopy revealed that the combination of miRNA-21 and cisplatin can induce autophagy in A549/DDP cells, and an increase in the number of autophagosomes and of autophagolysosomes was observed. Compared with miRNA-21 NC, when miRNA-21 sponge plus cisplatin interfered with A549/DDP cells, the cells showed necrotic characteristics, stained deeper, and disintegrated into small fragments that dispersed in the cytoplasm. Moreover, cell membranes ruptured, and the cells collapsed (**Figure 6B**).

Western blot showed that the expression of autophagy-related proteins LC3B and ATG7 were significantly increased (**Figure 6C**). The expression of apoptosis-related proteins cleasp-caspase 3 and Bax increased significantly, while the expression of Bcl-2 decreased (**Figure 6D**).

## Discussion

Despite the rapid development of modern medical technology, at present, the treatment of patients with advanced cancer is mainly a combination of surgery, radiotherapy, and chemotherapy. Among many clinical chemotherapy drugs, platinum is an important chemotherapeutic drug, and is widely used in various types of tumors. It is especially used in the clinical treatment of non-small cell lung cancer, and it can inhibit the recurrence and metastasis of tumors (23). However, after several rounds of chemotherapy, the tumor cells become multi-drug resistant to cisplatin, which brings great difficulties to clinical treatment and tumor recurrence.

As early as 1926, Warburg proposed that tumor cells can exhibit high glycolysis, including increased glucose consumption and lactic acid production, even under conditions of sufficient oxygen (24). With the continuous development of medicine, later studies have found that compared with healthy cells or tissues, the source of energy for tumor cells is not through the mitochondrial oxidative phosphorylation pathway, but depends on the glycolysis pathway, which further confirms the abnormality of mitochondrial metabolism (25). Studies have found that some key rate-limiting enzymes involved in the glycolytic pathway play an important role in the development of drug resistance in tumor cells. These enzymes include hexokinase (HK) in ovarian cancer (26) and phosphofructokinase (PFK) in colorectal cancer (27) and breast cancer (28). The expression of pyruvate kinase (PK) in cancer (29) and lactate dehydrogenase (LDH) in a variety of tumor cells (30–32) can often be elevated. In this study, the non-small cell lung cancer cell lines A549 and A549/DDP were used to investigate the effect of abnormal glucose metabolism on cisplatin resistance. Under normal oxygen conditions, the glucose consumption, lactate accumulation, and the ECAR in the cisplatin-resistant A549/DDP cell line increased compared to the cisplatin-sensitive A549 cell line. Detection by RNA-Seq and qRT-PCR showed that compared with the control group, A549/DDP cells showed increased PKM2 and LDHA mRNA expression level. Western blotting also detected an increase in the protein expression levels of PKM2 and LDHAs, while ELISA detected an increase in the enzyme activity levels of PK and LDH in A549/DDP cells. These data indicate that the non-small cell lung cancer cell line A549/DDP requires higher levels of glucose metabolism to adapt to the cisplatin-resistant phenotype.

The abnormal expression of some proto-oncogenes directly or indirectly affects the expression of glycolytic rate-limiting enzymes. For example, the proto-oncogene Myc plays an important regulatory role in cell transcription, translation, protein stability, and activity. Myc, when highly expressed, causes a variety of cancer characteristics. Research has found that the targeted regulation of miRNA and lncRNA downstream effectors promoted the expression of Myc target genes involved in glycolysis, namely GLUT1, LDHA, PKM2, and HK2. Ras oncogenes have long been a hot spot in oncology (33). In fact, RAS genes include HRAS, NRAS, and KRAS, of which KRAS is the most common. KRAS activates the downstream Raf/Mek/Erk cascade signaling pathway, which leads to the up-regulation of Myc transcription factors to promote glycolysis (34). In addition, KRAS protein can also interact with a variety of effector molecules such as MAPK, STAT, and PI3K, and can also regulate cell growth, proliferation, and metabolism (35). The proto-oncogene Src is a tyrosine kinase that can promote the proliferation, invasion, metastasis, and metabolism of cancer cells. Studies have found that Src inactivates PDH by directly phosphorylating PDHA1, reducing mitochondrial respiration and oxidative stress, leading to the Warburg effect (36). The PI3K/AKT signaling pathway acts as an intermediate linker in tumor cells, which can be regulated by the proto-oncogene Ras or Src, and can also regulate Myc and HIF-1α activity.

miRNA is a type of single-stranded RNA molecular fragment with a length of 21–25 nt, and it is widely present in eukaryotes. The combination of miRNA and mRNA through complementary pairing has a regulatory effect on the expression level of the complementary mRNA (37). It now stands confirmed that miRNA_S_ can regulate multiple biological functions of cells through the PI3K/AKT signaling pathway. For example, Cai (38) found that compared with normal cells and tissues, miR-27a is up-regulated in OA chondrocytes, but further studies have found that it decreases. The expression of miR-27a can induce autophagy and apoptosis in cells through the PI3K/AKT/mTOR signaling pathway. One study reported that the expression of miRNA-26a in cells is up-regulated after cerebral infarction in rats, which can activate PI3K/AKT, and the MAPK/ERK pathway up-regulates the expression of HIF-1a, which mediates the transcriptional activity of VEGF and promotes rat angiogenesis (39). Another study has also found that, compared with adjacent tissues and normal breast epithelial cell-line MCF-10A, the expression of miR-106b and miR-93 is up-regulated in breast cancer, and that miR-106b and miR-93 promote the migration, invasion, and proliferation of breast cancer cells through the PI3K/Akt pathway (40). However, only one study about miRNAS-regulating cell metabolism through the PI3K/AKT signaling pathway was searched. It was found that miR-181a-1 is highly expressed in hypertrophic chondrocytes. Further studies have found that miR-181a/b-1 regulates cell metabolism. The PTEN/PI3K/AKT axis enhances osteogenic differentiation, and mitochondrial metabolism promotes osteogenesis (41). In addition, miRNAs are also involved in the formation of drug resistance in many cancer cells (42–45). Studies have found that miRNA-21 is highly expressed in cisplatin-resistant non-small cell lung cancer A549/DDP cells. Down-regulating the expression of miRNA-21 can significantly reduce the multidrug resistance of A549/DDP cells (46). However, this observation has not provided a comprehensive explanation of the mechanism of action of miRNA-21. In this study, we compared cisplatin-sensitive A549 cells and cisplatin-resistant A549/DDP cells through qRT-PCR technology, and found that miRNA-21 is highly expressed in A549/DDP cells, which is consistent with the results of other researchers. In this study, it was also found that miR-21 inhibited the PI3K/AKT/mTOR/HIF-1α signaling pathway in A549/DDP cells, thereby inhibiting the expression of PKM2 and LDHA, and reducing the glycolysis level of A549/DDP cells, while the expression of c-Myc was not changed. A reduced PI3K/AKT/mTOR/HIF-1α signaling pathway suppressed the expression levels of key enzymes PKM2 and LDHA in the glycolysis pathway. The PI3K/AKT/mTOR signaling pathway plays an important role in regulating tumor cell proliferation, metastasis, apoptosis, and other physiological functions. The past view was that cell death was divided into cell necrosis and apoptosis based on whether or not it was controllable. However, in recent years, studies have found that cell necrosis is also subject to strict procedural control, and it is now called programmed necrosis (or necroptosis) (47). Programmed cell death is not dependent on caspase regulation but is regulated by the TNF-R1 and RIP1/RIP3-MLKL signaling pathways (48). One study has also reported that in certain cells, under certain conditions, cell apoptosis and necrosis can coexist and mutually transform (49). At the same time, the activation of autophagic lysosomes is also a downstream response mechanism of programmed necrosis. In our experimental results, when cisplatin and miRNA-21 sponge acted on A549/DDP cells alone, they tended to promote autophagy reaction to produce autophagosomes and autolysosomes. When cisplatin was combined with miRNA-21 sponge, it was observed with a fluorescence microscope, western blotting, and a transmission electron microscope that A549/DDP cells underwent apoptosis, autophagy, and cell necrosis at the same time. Whether cell necrosis is programmed necrosis needs further research.

In summary, these results indicate that miR-21 increases the cytotoxicity of cisplatin through the glucose metabolism reprogramming pathway and enhances cell death in cisplatin-resistant A549/DDP cells.

## Conclusions

This study suggests that the high expression of rate-limiting enzymes PKM2 and LDHA promoted glycolysis and induced the formation of cisplatin resistance in non-small cell lung cancer A549 cells. miRNA-21 combined with cisplatin inhibits glycolysis and induces cell death through the PI3K/AKT/mTOR/HIF-1α pathway, improves the cytotoxicity of cisplatin, and provides a theoretical basis for the treatment of cisplatin-resistant non-small cell lung cancer.

## Data Availability Statement

The datasets presented in this study can be found in online repositories. The names of the repository/repositories and accession number(s) can be found in the article/supplementary material.

## Author Contributions

CW was in charge of the study design, experiment modification,and drafting and finalizing of the paper. YS conducted themajority of the experiments and drafted a portion of the paper. QZ and RZ produced cytotoxic experiments. WL, JW and PP produced phosphorylation experiments. CL and HS offered crucial recommendations. All authors contributed to the article andapproved the submitted version.

## Conflict of Interest

The authors declare that the research was conducted in the absence of any commercial or financial relationships that could be construed as a potential conflict of interest.
